# Synthesis, Antimicrobial and Antiinflammatory Activity of 2,5-Disubstituted-1,3,4-oxadiazoles

**DOI:** 10.4103/0250-474X.40331

**Published:** 2008

**Authors:** G. Nagalakshmi

**Affiliations:** Department of Pharmaceutical Chemistry, The Erode College of Pharmacy, Vallipurathanpalayam post, Erode - 638 112, India

**Keywords:** 1,3,4-oxadiazole, 2,5-disubstituted-1,3,4-oxadiazole, antimicrobial agents, 4-methoxybenzohydrazide, antiinflammatory activity

## Abstract

In the present study, 2,5-disubstituted-1,3,4-oxadiazoles (3a-o) have been synthesized by the condensation of 4-methoxybenzohydrazide (1) with different aromatic acids (2a-o) in presence of phosphoryl chloride. The structural assignment of this compound (3a-o) has been made on the basis of elemental analysis, UV, IR, ^1^H NMR and mass spectral data. The synthesized compounds were screened for their *in vitro* growth inhibiting activity against different strains of bacteria and fungi viz., *Staphylococcus aureus, Bacillus subtilis, Bacillus megaterium, Escherichia coli, Pseudomonas aeruginosa, Shigella dysenteriae, Candida albicans, Aspergillus niger* and *Aspergillus flavus* were compared with the standard antibiotics such as chloramphenicol (50 μg/ml) and griseofulvin (50 μg/ml) using well agar diffusion technique. Compounds 3e, 3g, 3h and 3m exhibits highest antibacterial activity and compounds 3d, 3g and 3h showed better antifungal activity. The synthesized compounds (3a-o) were screened for their *in vitro* antiinflammatory activity against carrageenan-induced rat paw oedema. Compounds 3f and 3i were found to be most active compound of this series, which shows 46.42% and 50% inflammation inhibitory activity, whereas standard drug phenylbutazone exhibit 53.57% antiinflammatory activity at a dose of 50 mg/kg po.

2,5-Disubstituted-1,3,4-oxadiazoles have been found to exhibit diverse biological activities such as antibacterial^1^, antiHIV^1^, antifungal^2^, genotoxic^2^, antitubercular^3^, virucidal^4^, antimalarial^5^, insecticidal^6^, herbicidal^7^, analgesic^8^, antiinflammatory^9^, muscle relaxants^10^, anticonvulsant^11^, sedative, hypnotic^12^, anticancer^13^ and lipid peroxidation inhibitor^14^. Aryl alkanoic acids provide one of the fascinating classes of compounds recognized for various pharmacological activities like antipyretic, analgesic and anti-inflammatory^15^, used extensively in the symptomatic treatment of rheumatic fever, arthritis^16^ (rheumatoid, osteo and jaundice arthritis), myocardial infarctions and management of primary dysmenorrhea^17^.

The major side effects in the use of aryl alkanoic acids is their gastric irritancy, which is partly due to the corrosive nature of carboxylic acid group present in them. In order to reduce or mask the side effects of carboxylic moiety we planned to synthesize various 2,5-disubstituted-1,3,4-oxadiazole via the condensation of 4-methoxybenzohydrazide with various aromatic acids in presence of phosphoryl chloride respectively in the hope of getting potent biodynamic agents and evaluate their antimicrobial and antiinflammatory activity.

## MATERIALS AND METHODS

The identification and purity of the products were checked by TLC (Merck Silica-60F_254_) with Ethyl acetate: acetone (9:1) using iodine vapours and UV light as detecting agents and the Rf value were given below. Melting points were measured on open capillaries in a liquid paraffin bath and are uncorrected. The absorbance maxima (λmax) were determined on a Systronics UV-Vis double beam spectrophotometer (2201) in ethanol. IR Spectra were taken on a Perkin Elmer Spectrum RX I, FTIR Spectrophotometer using potassium bromide pellets. ^1^H NMR spectra were recorded in DMSO-d_6_ on AMX-400, NMR spectrometer using TMS as an internal standard (chemical shift in δ ppm). FAB mass spectra were taken out on a JEOL SX102/DA-6600 mass spectrometer using Argon/Xenon (6 kV, 10 mA) as the FAB gas. Elemental analysis was obtained on a Carlo Erba 1108 Heraeus elemental analyzer. All the chemicals used were of synthetic and AR grade and were procured from Alfa Aesar (4-methoxybenzohydrazide), USA, S.D. Fine Chem. Ltd and Merck, Mumbai, India.

### General procedure for the synthesis of 2,5-disubstituted-1,3,4-oxadiazoles (3a-o):

A mixture of different aromatic acid(s) (0.01 mol) with 4-methoxybenzohydrazide (1.6617 g, 0.01 mol) in phosphoryl chloride (15 ml) was refluxed over a steam bath for 5-6 h. The progress of the reaction was monitored by TLC (Merck Silica-60F_254_) using ethyl acetate: acetone (9:1) as eluent. The reaction mixture was cooled and poured on to crushed ice (∼200 g) with continuous stirring. The solid mass separated was neutralized with sodium bicarbonate solution (10% w/v). The resulting solid thus obtained was collected by filtration, washed well with cold water, dried in vacuum and recrystallized from absolute ethanol (95%) and analyzed. Adopting the above procedure fifteen different 2,5-disubstituted-1,3,4-oxadiazoles (3a-o) were synthesized and their characterization data are presented in Table 1. Yield and melting point of the product(s) were determined and summarized below.

**TABLE 1:** PHYSICAL AND ANALYTICAL DATA OF 2,5-DISUBSTITUTED-1,3,4- OXADIAZOLES (3a-o)

Compounds	R	Mol. Formula Mol. weight	Elemental analysis found (calcd) %		
			
			C	H	N
3a	C_6_H_5_	C_15_H_12_N_2_O_2_/252.26	71.37 (71.42)	4.77 (4.79)	11.06 (11.10)
3b	4-CH_3_C_6_H_4_	C_16_H_14_N_2_O_2_/266.29	72.13 (72.16)	5.26 (5.30)	10.49 (10.52)
3c	CH = CH-C_6_H_5_	C_17_H_14_N_2_O_2_/278.30	73.32 (73.37)	5.04 (5.07)	10.02 (10.07)
3d	4-NH_4_C_6_H_4_	C_15_H_13_N_3_O_2_/267.28	67.36 (67.40)	4.86 (4.90)	15.70 (15.72)
3e	4-NO_2_C_6_H_4_	C_15_H_11_N_3_O_4_/297.26	60.58 (60.61)	3.70 (3.73)	14.10 (14.14)
3f	3,5-(NO_2_)_2_C_6_H_3_	C_15_H_10_N_4_O_6_/342.26	52.61 (52.64)	2.90 (2.94)	16.34 (16.37)
3g	2,4-(NO_2_)_2_C_6_H_3_NHC_6_H_4_	C_21_H_15_N_5_O_6_/433.37	58.18 (58.20)	3.45 (3.49)	16.12 (16.16)
3h	2-NO_2_C_6_H_4_NHC_6_H_4_	C_21_H_16_N_4_O_4_/388.37	64.91 (64.94)	4.12 (4.15)	14.38 (14.43)
3i	C_6_H_5_CONHC_6_H_4_	C_22_H_17_N_3_O_3_/371.38	71.14 (71.15)	4.58 (4.61)	10.42 (10.44)
3j	4-OHC_6_H_4_	C_15_H_12_N_2_O_3_/268.26	67.13 (67.16)	4.49 (4.51)	11.28 (11.31)
3k	3,4,5-(OCH_3_)3C_6_H_2_	C_18_H_18_N_2_O_5_/342.34	63.12 (63.15)	5.28 (5.30)	8.18 (8.18)
3l	C_5_H_4_N	C_14_H_11_N_3_O_2_/253.25	66.38 (66.40)	4.37 (4.38)	16.56 (16.59)
3m	2,4-(OH)_2_C_6_H_3_	C_15_H_12_N_2_O_4_/284.26	63.35 (63.38)	4.24 (4.25)	9.84 (9.85)
3n	3-NH_2_C_6_H_4_	C_15_H_13_N_3_O_2_/267.28	67.40 (67.40)	4.89 (4.90)	15.70 (15.72)
3o	2-OH3-CH_3_C_6_H_3_	C_16_H_14_N_2_O_3_/282.29	68.04 (68.07)	4.98 (5.00)	9.89 (9.92)

### 2-(4-methoxyphenyl)-5-phenyl-1,3,4-oxadiazole (3a):

Yield: 69.37% (1.75 g); mp: 221°; Rf value: 0.69; UV (λmax, nm): 348.3; IR (KBr, cm^−1^): 3027 (aromatic C-H), 1602, 1493, 1456 (aromatic C = C), 1650 (C = N), 1210 (asymmetric C-O-C), 1028 (symmetric C-O-C), 2962 (methyl C-H, γas CH_3_), 2872 (methyl C-H, γs CH_3_); ^1^H NMR (DMSO-d_6_, δ ppm): 6.87-7.0 (m, 4H, Ar-H), 7.21-7.38 (m, 5H, Ar-H), 3.74 (s, 3H, Ar-OCH_3_); MS (FAB) m/z: 252 (M^+^), 253 (M^+^ + 1, 100%) for C_15_H_12_N_2_O_2_.

### 2-(4-methoxyphenyl)-5-(4-methylphenyl)-1,3,4-oxadiazole (3b):

Yield: 78.86% (2.1 g); mp: 229°; Rf value: 0.84; UV (λmax, nm): 276.2; IR (KBr, cm^−1^): 3040 (aromatic C-H), 1595, 1499, 1472 (aromatic C = C), 1640 (C = N), 1250 (asymmetric C-O-C), 1032 (symmetric C-O-C), 2950 (methyl C-H, γas CH_3_), 2859 (methyl C-H, γs CH_3_), 750 (out-of-plane aromatic C-H bend); ^1^H NMR (DMSO-d_6_, δ ppm): 6.87-7.10 (m, 4H, Ar-H), 7.27-7.48 (m, 4H, Ar-H), 2.37 (s, 3H, Ar-CH_3_), 3.76 (s, 3H, Ar-OCH_3_); MS (FAB) m/z: 266 (M^+^), 267 (M^+^ + 1, 100%) for C_16_H_14_N_2_O_2_.

### 2-(4-methoxyphenyl)-5-(2-phenylvinyl)-1,3,4-oxadiazole (3c):

Yield: 70.09% (1.95 g); mp: 231°; Rf value: 0.78; UV (λmax, nm): 267.2; IR (KBr, cm^−1^): 3055 (aromatic C-H), 1604, 1493, 1464 (aromatic C = C), 1668 (C = N), 2972 (methyl C-H, γas CH_3_), 2840 (methyl C-H, γs CH_3_), 1249 (asymmetric C-O-C), 1040 (symmetric C-O-C), 3048 (alkene C-H), 1640 (C = C, alkene), 991 (out-of-plane alkene C-H bend); ^1^H NMR (DMSO-d_6_, δ ppm): 6.82-7.12 (m, 4H, Ar-H), 7.21-7.38 (m, 5H, Ar-H), 3.9 (s, 3H, Ar-OCH_3_), 4.82-5.92 (d, 2H, CH = CH); MS (FAB) m/z: 278 (M^+^), 279 (M^+^ + 1, 100%) for C_17_H_14_N_2_O_2_.

### 4-[5-(4-methoxyphenyl)-1,3,4-oxadiazol-2-yl]aniline (3d):

Yield: 80.44% (2.15 g); mp: 202°; Rf value: 0.82; UV (λmax, nm): 278.2; IR (KBr, cm^−1^): 3048 (aromatic C-H), 1598, 1494, 1474 (aromatic C = C), 1654 (C = N), 1252 (asymmetric C-O-C), 1047 (symmetric C-O-C), 3510 (N-H, aromatic primary amine, asymmetric), 3421 (N-H, aromatic primary amine, symmetric), 1292 (C-N, aromatic primary amine), 2965 (methyl C-H, γas CH_3_), 2874 (methyl C-H, γs CH_3_); ^1^H NMR (DMSO-d_6_, δ ppm): 6.85-7.07 (m, 4H, Ar-H), 7.29-7.48 (m, 4H, Ar-H), 3.82 (s, 3H, Ar-OCH_3_), 4.48 (s, 2H, NH_2_); MS (FAB) m/z: 267 (M^+^), 268 (M^+^ + 1, 100%) for C_15_H_13_N_3_O_2_.

### 2-(4-methoxyphenyl)-5-(4-nitrophenyl)-1,3,4-oxadiazole (3e):

Yield: 65.60% (1.95 g); mp: 235°; Rf value: 0.69; UV (λmax, nm): 273.2; IR (KBr, cm^−1^): 3054 (aromatic C-H), 1603, 1498, 1470 (aromatic C = C), 1646 (C = N), 1247 (asymmetric C-O-C), 1046 (symmetric C-O-C), 1523 (asymmetric ArNO_2_, NO_2_), 1347 (symmetric ArNO_2_, NO_2_), 852 (C-N, ArNO_2_), 2958 (methyl C-H, γas CH_3_), 2843 (methyl C-H, γs CH_3_); ^1^H NMR (DMSO-d_6_, δ ppm): 6.83-7.0 (m, 4H, Ar-H), 7.44-7.48 (m, 4H, Ar-H), 3.81 (s, 3H, Ar-OCH_3_); MS (FAB) m/z: 297 (M^+^, 100%) for C_15_H_11_N_3_O_4_.

### 2-(3,5-dinitrophenyl)-5-(4-methoxyphenyl)-1,3,4-oxadiazole (3f):

Yield: 82.39% (2.82 g); mp: 258°; Rf value: 0.78; UV (λmax, nm): 282.0; IR (KBr, cm^−1^): 3048 (aromatic C-H), 1609, 1492, 1462 (aromatic C = C ring), 1648 (C = N), 1258 (asymmetric C-O-C), 1052 (symmetric C-O-C), 1538 (asymmetric ArNO_2_, NO_2_), 1350 (symmetric ArNO_2_, NO_2_), 856 (C-N, ArNO_2_), 2963 (methyl C-H, γas CH_3_), 2874 (methyl C-H, γs CH_3_); ^1^H NMR (DMSO-d_6_, δ ppm): 6.85-7.17 (m, 4H, Ar-H), 7.31-7.52 (m, 3H, Ar-H), 3.78 (s, 3H, Ar-OCH_3_); MS (FAB) m/z: 342 (M^+^, 100%) for C_15_H_10_N_4_O_6._

### N-{4-[5-(4-methoxyphenyl)-1,3,4-oxadiazol-2-yl]phenyl}-2,4-dinitroaniline (3g):

Yield: 87.68% (3.8 g); mp: 249°; Rf value: 0.69; UV (λmax, nm): 268.4; IR (KBr, cm^−1^): 3045 (aromatic C-H), 1596, 1496, 1472 (aromatic C = C), 1672 (C = N), 1260 (asymmetric C-O-C), 1050 (symmetric C-O-C), 1530 (asymmetric ArNO_2_, NO_2_), 1348 (symmetric ArNO_2_, NO_2_), 854 (C-N, ArNO_2_), 3332 (N-H, aromatic secondary amine), 1310 (C-N, secondary aromatic amine), 2950 (methyl C-H, γas CH_3_), 2836 (methyl C-H, γs CH_3_); ^1^H NMR (DMSO-d_6_, δ ppm): 6.86-7.0 (m, 4H, Ar-H), 7.21-7.48 (m, 7H, Ar-H), 3.80 (s, 3H, Ar-OCH_3_), 2.18 (s, 1H, NH); MS (FAB) m/z: 433 (M^+^), 434 (M^+^ + 1, 100%) for C_21_H_15_N_5_O_6_.

### N-{4-[5-(4-methoxyphenyl)-1,3,4-oxadiazol-2-yl]phenyl}-2-nitroaniline (3h):

Yield: 75.18% (2.92 g); mp: 224°; Rf value: 0.75; UV (λmax, nm): 285.2; IR (KBr, cm^−1^): 3038 (aromatic C-H), 1596, 1492, 1458 (aromatic C = C), 1636 (C = N), 1246 (asymmetric C-O-C), 1046 (symmetric C-O-C), 1520 (asymmetric ArNO_2_, NO_2_), 1342 (symmetric ArNO_2_, NO_2_), 854 (C-N, ArNO_2_), 3332 (N-H, aromatic secondary amine), 1298 (C-N, secondary aromatic amine), 2964 (methyl C-H, γas CH_3_), 2870 (methyl C-H, γs CH_3_); ^1^H NMR (DMSO-d_6_, δ ppm): 6.89-7.14 (m, 4H, Ar-H), 7.20-7.52 (m, 8H, Ar-H), 3.78 (s, 3H, Ar-OCH_3_), 2.21 (s, 1H, NH); MS (FAB) m/z: 388 (M^+^, 100%) for C_21_H_16_N_4_O_4_.

### N-{4-[5-(4-methoxyphenyl)-1,3,4-oxadiazol-2-yl]phenyl}benzamide (3i):

Yield: 71.35% (2.65 g); mp: 242°; Rf value: 0.68; UV (λmax, nm): 288.0; IR (KBr, cm^−1^): 3065 (aromatic C-H), 1607, 1496, 1465 (aromatic C = C), 1654 (C = N), 1245 (asymmetric C-O-C), 1040 (symmetric C-O-C), 3430 (N-H, secondary amide), 1644 (C = O, secondary amide), 2968 (methyl C-H, γas CH_3_), 2875 (methyl C-H, γs CH_3_); ^1^H NMR (DMSO-d_6_, δ ppm): 6.87-7.10 (m, 4H, Ar-H), 7.18-7.48 (m, 9H, Ar-H), 3.86 (s, 3H, Ar-OCH_3_), 8.51 (s, 1H, CONH); MS (FAB) m/z: 371 (M^+^), 372 (M^+^ + 1, 100%) for C_22_H_17_N_3_O_3_.

### 4-[5-(4-methoxyphenyl)-1,3,4-oxadiazol-2-yl]phenol (3j):

Yield: 75.27% (2.125 g); mp: 279°; Rf value: 0.79; UV (λmax, nm): 270.0; IR (KBr, cm^−1^): 3030 (aromatic C-H), 3598 (O-H), 1224 (C-O), 1605, 1495, 1465 (aromatic C = C), 1645 (C = N), 1252 (asymmetric C-O-C), 1049 (symmetric C-O-C), 2960 (methyl C-H, γas CH_3_), 2869 (methyl C-H, γs CH_3_), 750 (out-of-plane aromatic C-H bend), 1361 (in-plane O-H bend); ^1^H NMR (DMSO-d_6_, δ ppm): 6.87-7.13 (m, 4H, Ar-H), 3.52 (s, 3H, OCH_3_), 7.17-7.42 (m, 4H, Ar-H), 10.44 (s, 1H, Ar-OH); MS (FAB) m/z: 268 (M^+^), 269 (M^+^ + 1, 100%) for C_15_H_12_N_2_O_3_.

### 2-(4-methoxyphenyl)-5-(3,4,5-trimethoxyphenyl)-1,3,4-oxadiazole (3k):

Yield: 77.48% (2.65 g); mp: 252°; Rf value: 0.81; UV (λmax, nm): 310.2; IR (KBr, cm^−1^): 3045 (aromatic C-H), 1219 (C-O), 1608, 1492, 1459 (aromatic C = C), 1634 (C = N), 1242 (asymmetric C-O-C), 1021 (symmetric C-O-C), 2963 (methyl C-H, γas CH_3_), 2872 (methyl C-H, γs CH_3_); ^1^H NMR (DMSO-d_6_, δ ppm): 6.37-6.92 (m, 2H, Ar-H), 7.18-7.42 (m, 4H, Ar-H), 3.9 (s, 9H, OCH_3_); MS (FAB) m/z: 342 (M^+^), 343 (M^+^ + 1, 100%) for C_18_H_18_N_2_O_5_.

### 3-[5-(4-methoxyphenyl)-1,3,4-oxadiazol-2-yl]pyridine (3l):

Yield: 73.05% (1.85 g); mp: 240°; Rf value: 0.79; UV (λmax, nm): 285.4; IR (KBr, cm^−1^): 3040 (aromatic C-H), 1601, 1492, 1473 (aromatic C = C), 1642 (C = N), 1251 (asymmetric C-O-C), 1045 (symmetric C-O-C), 2960 (methyl C-H, γasy CH_3_), 2869 (methyl C-H, γsy CH_3_), 748 (out-of-plane aromatic C-H bend), 690 (out-of-plane ring C = C bend); ^1^H NMR (DMSO-d_6_, δ ppm): 6.92-7.12 (m, 4H, Ar-H), 7.70-7.75 (d, 4H, pyridyl), 3.85 (s, 3H, OCH_3_); MS (FAB) m/z: 239 (M^+^, 100%), 240 (M + 1)^+^ for C_14_H_11_N_3_O_2_.

### 4-[5-(4-methoxyphenyl)-1,3,4-oxadiazol-2-yl]benzene-1,3-diol (3m):

Yield: 68.59% (1.95 g); mp: 287°; Rf value: 0.80; UV (λmax, nm): 281.6; IR (KBr, cm^−1^): 3038 (aromatic C-H), 3602 (O-H), 1224 (C-O), 1606, 1446, 1466 (aromatic C = C), 1645 (C = N), 1249 (asymmetric C-O-C), 1035 (symmetric C-O-C), 2965 (C-H, γas CH_3_), 2876 (C-H, γs CH_3_), 1224 (C-O), 780 (out-of-plane aromatic C-H bend), 689 (out-of-plane ring C = C bend); ^1^H NMR (DMSO-d_6_, δ ppm): 6.87-7.11 (m, 4H, Ar-H), 7.17-7.38 (m, 3H, Ar-H), 10.7 (s, 2H, OH), 3.86 (s, 3H, OCH_3_); MS (FAB) m/z: 284 (M^+^), 285 (M^+^ + 1, 100%) for C_15_H_12_N_2_O_4_.

### 3-[5-(4-methoxyphenyl)-1,3,4-oxadiazol-2-yl]aniline (3n):

Yield: 71.08% (1.90 g); mp: 217°; Rf value: 0.81; UV (λmax, nm): 292.0; IR (KBr, cm^−1^): 3045 (aromatic C-H), 1602, 1497, 1470 (aromatic C = C), 1647 (C = N), 1250 (asymmetric C-O-C), 1041 (symmetric C-O-C), 3522 (N-H, aromatic primary amine, asymmetric), 3416 (N-H, primary amine, symmetric), 2968 (C-H, γas CH_3_), 2877 (C-H, γs CH_3_), 1294 (C-N str, aromatic primary amine); ^1^H NMR (DMSO-d_6_, δ ppm): 6.92-7.21 (m, 4H, Ar-H), 7.27-7.52 (m, 4H, Ar-H), 3.81 (s, 3H, OCH_3_), 4.41 (s, 2H, NH_2_); MS (FAB) m/z: 267 (M^+^), 268 (M^+^ + 1, 100%) for C_15_H_13_N_3_O_2_.

### 2-[5-(4-methoxyphenyl)-1,3,4-oxadiazol-2-yl]-3-methylphenol (3o):

Yield: 74.39% (2.1 g); mp: 261°; Rf value: 0.72; UV (λmax, nm): 291.0; IR (KBr, cm^−1^): 3050 (aromatic C-H), 3606 (O-H), 1226 (C-O), 1595, 1499, 1470 (aromatic C = C), 1652 (C = N), 1247 (asymmetric C-O-C), 1040 (symmetric C-O-C), 2962 (methyl C-H, γas CH_3_), 2872 (methyl C-H, δs CH_3_), 1378 (C-H bend, ds CH_3_), 1362 (in-plane O-H bend), 692 (out-of-plane ring C = C bend); ^1^H NMR (DMSO-d_6_, δ ppm): 6.42-6.92 (m, 3H, Ar-H), 6.97-7.21 (m, 4H, Ar-H), 10.28 (s, 1H, OH), 2.42 (s, 3H, Ar-CH_3_) 3.82 (s, 3H, OCH_3_); MS (FAB) m/z: 282 (M^+^), 283 (M^+^ + 1, 100%) for C_16_H_14_N_2_O_3_.

### Screening for antimicrobial activity:

The antimicrobial activity of all the newly synthesized compounds were determined by well plate method^18^ in nutrient agar (Hi-Media) was used for antibacterial activity and Sabouraud dextrose agar (SDA) (Hi-Media) was used for antifungal activity. The bacterial strain used were *Bacillus subtilis* (ATCC 6633), *Staphylococcus aureus* (ATCC 25923) and *Bacillus megaterium* (ATCC 1327) for gram positive and *Escherichia coli* (ATCC 25922), *Pseudomonas aeruginosa* (ATCC 27853) and *Shigella dysenteriae* (ATCC 13313) for gram negative and for fungal strain viz., *Candida albicans* (ATCC 10231), *Aspergillus niger* (ATCC 16404) and *Aspergillus flavus* (ATCC 22547).

The compounds were tested at a concentration of 100 μg/ml (or) 0.00001 nanomoles were prepared in dimethylformamide (DMF). The petridishes used for antibacterial screening were incubated at 37 ± 1° for 24 h, while those used for antifungal activity were incubated at 28° for 48-72 h. The diameters of zone of inhibition (mm) surrounding each of the wells were recorded.

The results were compared to chloramphenicol (50 μg/ml (or) 0.000005 nanomoles) and griseofulvin (50 μg/ml (or) 0.000005 nanomoles) for antibacterial and antifungal activity. The antibacterial and antifungal screening results were presented in Table 2 and Table 3.

**TABLE 2:** ANTIBACTERIAL SCREENING RESULTS OF COMPOUNDS (3a-o)

Compounds	Antibacterial activity					
	
	Zone of inhibition (mm)					
	
	*B. subtilis*	*Staph. aureus*	*B. megaterium*	*E. coli*	*P. aeruginosa*	*S. dysenteriae*
3a	10	12	13	14	12	12
3b	20	20	17	19	16	15
3c	11	15	13	13	13	10
3d	16	16	15	20	15	14
3e	23	19	21	22	17	15
3f	14	14	15	14	13	12
3g	22	17	18	24	17	20
3h	21	14	22	22	20	16
3i	16	14	15	17	14	14
3j	24	15	16	20	20	12
3k	15	12	15	17	12	14
3l	12	13	12	13	14	11
3m	21	16	17	22	19	18
3n	20	20	15	20	15	12
3o	17	16	16	14	14	15
Chloramphenicol	23	22	20	26	24	22

**TABLE 3:** ANTIFUNGAL SCREENING RESULTS OF COMPOUNDS (3a-o)

Compounds	Antifungal activity		
	
	Zone of inhibition (mm)		
	
	*Candida albicans*	*Aspergillus niger*	*Aspergillus flavus*
3a	17	16	13
3b	14	14	15
3c	13	11	16
3d	18	19	18
3e	14	16	14
3f	15	12	11
3g	21	19	19
3h	20	19	18
3i	15	16	18
3j	17	17	14
3k	16	15	13
3l	13	14	12
3m	18	17	15
3n	14	14	12
3o	18	16	18
Griseofulvin (50μg/ml)	21	21	19

### Acute toxicity study:

Acute toxicity study was carried out by “Stair case” method^19^. Swiss mice of either sex were injected with a particular dose, say 100 mg/kg and observed for a period of 24 h for any mortality. The subsequent doses are then increased by a factor 1.5 if the dose was tolerated, and decreased by a factor 0.7 if it was lethal. The LD_50_ of the drug was found to be 500 mg/kg body wt. One tenth of this dose was selected as the therapeutic dose for evaluation (i.e. 50 mg/kg).

### Antiinflammatory activity against carrageenan-induced rats paws oedema:

Antiinflammatory activity was determined by carrageenan-induced rat paw method of winter *et al.*^20^. Male Wistar rats (120-150 g) was used for the experiment. They were fed with standard pellet diet and water was given *ad libitum*. The animals were acclimatized for one week under laboratory conditions before performing the test. They were housed in polypropylene cages under standard conditions (30 ± 1°, 12/12 h light/dark cycles on 60-70% RH). The standard groups received phenylbutazone 50 mg/kg body weight po, suspended in 1% w/v of carboxymethylcellulose (CMC) in distilled water. The test group received synthesized compounds (3a-o) (50 mg/kg body weight po, suspended in 1% w/v of CMC in water). The control group received corresponding amount of vehicle (1% w/v of CMC). All the test compounds and standard drug were administered 30 min prior to carrageenan injection. The antiinflammatory activity of synthesized compounds (3a-o) was carried out in Periyar College of Pharmaceutical Sciences for Girls, Tiruchirappalli 21, Tamilnadu. Before performing these experiments, ethical clearance was obtained from Institutional Animal Ethics Committee and conducted according to the Indian National Science Academy guidelines for the use and care of experimental animals (CPCSEA Reg No. 418).

Acute edema was induced in the right hind paw of rats by injecting 0.1 ml of freshly prepared 1% w/v of aqueous solution of carrageenan (Sigma, USA) in the subplanter region of right hind paw. After the carrageenan injection the paw volume was measured before and after 1, 2 and 3 h by plethysmometer (UGO-Basile, Italy). The difference between the left and right paw was taken as a measure of oedema. Any significant reduction in the volume of the paw compared to the control group was considered as anti-inflammatory response^21^. Percent inhibition of inflammation after 3 h was calculated by applying Newbould formula. % inhibition = 100 [1 – a – x/b – y], where, x = mean paw volume of rats before the administration of carrageenan injection in the test and the standard groups, y = mean paw volume of rats before the administration of carrageenan injection in the control group, a = mean paw volume of rats after the administration of carrageenan and test compound or standard compound, b = mean paw volume of rats after the administration of carrageenan injection in control group. The results are presented in Table 4.

**TABLE 4:** ANTIINFLAMMATORY ACTIVITY OF 2,5- DISUBSTITUTED-1,3,4-OXADIAZOLES (3a-o)

Compounds	Normal paw volume (x)	Paw oedema 3 h after Carrageenan injected (a)	% inhibition of oedema (1 – a – x/ b – y) × 100
3a	0.71 ± 0.04	0.93 ± 0.05	21.42
3b	0.68 ± 0.02	0.85 ± 0.02	39.28
3c	0.69 ± 0.02	0.87 ± 0.02	35.71
3d	0.66 ± 0.04	0.82 ± 0.03	42.86
3e	0.72 ± 0.03	0.95 ± 0.03	17.85
3f	0.68 ± 0.02	0.83 ± 0.04	46.42
3g	0.70 ± 0.03	0.86 ± 0.03	42.86
3h	0.69 ± 0.02	0.89 ± 0.03	28.57
3i	0.66 ± 0.04	0.80 ± 0.03	50.00
3j	0.675 ± 0.02	0.832 ± 0.04	43.96
3k	0.712 ± 0.04	0.940 ± 0.03	18.46
3l	0.660 ± 0.04	0.836 ± 0.04	37.21
3m	0.670 ± 0.02	0.841 ± 0.02	39.10
3n	0.710 ± 0.04	0.92 ± 0.04	25.00
3o	0.694 ± 0.02	0.852 ± 0.02	43.46
Control	0.69 ± 0.02 (y)	0.97 ± 0.03 (b)	-
Phenyl- butazone	0.70 ± 0.04	0.83 ± 0.03	53.57

***P<0.001, *P vs.* standard mean ± SEM

## RESULTS AND DISCUSSION

**Scheme 1:**
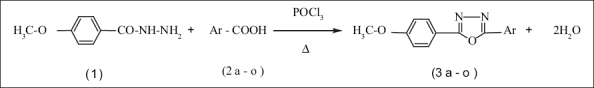
Synthetic route for the preparation of novel 2,5-disubstituted-1,3,4-oxadiazoles 3a-o.

2,5-Disubstituted-1,3,4-oxadiazole (3a-o) was synthesized by the condensation of 4-methoxybenzohydrazide with various aromatic acids in presence of phosphoryl chloride (Scheme 1). The physical and analytical data of the compounds (3a-o) were collected and presented in Table 1. The yields of 3a-o fall in the range of 66-88%. The spectral (IR, ^1^H NMR and MS) and analytical data are in good agreement with their structures.

In the toxicity study, LD_50_ of the drug was found to be 500 mg/kg body wt. The therapeutic dose of the drug is considered as 1/10^th^ of the LD_50_ value. Screening results of antimicrobial activity reveal (Table 2) that the known standard antibiotics chloramphenicol (50 μg/ml (or) 0.000005 nanomoles) and griseofulvin (50 μg/ml (or) 0.000005 nanomoles) showed zone of inhibition at 20-26 mm and 19-21 mm against bacterial and fungal strains. Compound 3e and 3j displayed better activity against *Bacillus subtilis*, while the compound 3b, 3e and 3n showed maximum activity against *Staphylococcus aureus*. Compound 3e and 3h exhibited significant activity against *Bacillus megaterium*. Compound 3e, 3g, 3h and 3m were highly active against *Escherichia coli* whereas compound 3g, 3h, 3j and 3m displayed moderate activity against *Pseudomonas aeruginosa* and *Shigella dysenteriae*. Compound 3g showed better antifungal activity against *Candida albicans* and compound 3d, 3g, 3h, 3i and 3o displayed moderate activity against *Aspergillus niger* and *Aspergillus flavus* when compared to standard.

The results in Table 4 indicate that the compounds 3f and 3i are active (*p* < 0.001) with the standard. Moreover, compounds 3d, 3g, 3j and 3o show less significant anti-inflammatory activity (*p* < 0.01). Carrageenan-induced paw edema was taken as a prototype of exudative phase of inflammation. The development of edema has been described as biphasic. The initial phase is due to the release of histamine, serotonins, 5-hydroxy tryptamine and kinins in the first hour after injection of carrageenan. More pronounced second phase is related to the release of prostaglandin^22^–^24^ like substances in 2-3 h. Hence, the significant anti-inflammatory effect may be due to an inhibitory effect exerted predominantly on the mediators of inflammation induced by phlogogenic stimuli.
